# COVID-19 and the Demand for Online Grocery Shopping: Empirical Evidence from the Netherlands

**DOI:** 10.1007/s10645-021-09389-y

**Published:** 2021-07-07

**Authors:** Barbara Baarsma, Jesse Groenewegen

**Affiliations:** 1 University of Amsterdam, Amsterdam, Netherlands; 2 Utrecht University, Utrecht, Netherlands

**Keywords:** Consumer behavior, COVID-19, Online grocery shopping, Food consumption, D12, L81, R22

## Abstract

There has been a pronounced increase in online shopping since the start of the COVID-19 pandemic. We study the effect of the pandemic on demand for online grocery shopping specifically, using municipality-level data from a Dutch online supermarket. We find that an additional hospital admission increased app traffic by 7.3 percent and sales per order by 0.31 percent. Local hospital admissions do not correlate with the variety of groceries ordered, but online search behavior does, suggesting that hoarding behavior is driven by the general perception and impact of the virus rather than local conditions. Local COVID-19 conditions also have different effects in urban versus non-urban municipalities, with local hospital admissions increasing app traffic in urban areas but lowering sales per order as compared to non-urban areas. It remains to be seen whether the demand for online grocery shopping will permanently increase as a result of the COVID-19 pandemic.

**Supplementary Information:**

The online version contains supplementary material available at 10.1007/s10645-021-09389-y.

## Introduction

The COVID-19 pandemic initially had a significant negative effect on household consumption worldwide (Baker et al., 2020; Hacioglu et al., 2020; Kim et al., 2020; Piyapromdee & Spittal, 2020), but there were exceptions to this decline in consumption.

First, people started to buy more online (Chen, et al. (2021); Relihan et al., 2020). This trend, which already existed before the pandemic, has since accelerated. In the European Union, almost six in ten consumers aged 16–75 bought goods or services online in 2017, compared to only three in ten in 2007 (Statistics Netherlands, 2018). In the Netherlands, which has a high proportion of residents with internet access, 79 percent shopped online in 2017. That percentage rose to 81 percent in 2019 (Eurostat, 2020), and is likely to further increase after the pandemic (United Nations Conference on Trade & Development, 2020).

Second, food consumption patterns have shifted sharply (Cavallo, 2020).^1^ Dutch consumers spent less on most consumer goods in the first 9 months of the pandemic, but, just like in many other countries, spent more on daily groceries than before. According to Statistics Netherlands, sales of non-perishable foods, such as canned foods, frozen vegetables, rice and pasta, increased strongly during the national lockdown that was in place in the Netherlands in March and April 2020. Meanwhile, restaurants and cafes were closed and people mainly worked from home, meaning that expenditures in company restaurants, canteens, and ‘on the go’ shops, such as those at gas stations and at train stations, were much lower.

During the first lockdown these two developments coincided and online expenditures on daily groceries increased markedly, as panic buying led to empty shelves and anxious consumers sought to avoid crowded supermarkets.

This sudden shift to online grocery shopping is a relatively new development globally but also in the Netherlands. Until 2006, groceries were hardly bought online; in 2017 the share of consumers in the Netherlands who shopped for groceries online was the highest in the EU at almost 30 percent (Statistics Netherlands, 2018). Up-to-date figures on developments during the pandemic are not yet available for the Netherlands, but online sales of food products also increased sharply in other countries during the pandemic (United Nations Conference on Trade & Development, 2020).

In the Netherlands, supermarkets and other food stores have remained open during the pandemic. It is therefore likely that people have started to avoid physical stores because of fears of virus transmission (Andersen et al., 2020; Watanabe & Omori, 2020; Yilmazkuday, 2020). Golec et al. (2020) show for the Netherlands that the aggregated expenditure on online groceries is indeed higher in more severely affected municipalities. Grashuis et al. (2020) find that in the United States in localities where COVID-19 is spreading at an increasing rate, consumers are generally less willing to shop inside grocery stores and move to online shopping instead.

In this paper, we analyze the link between the pandemic and online grocery shopping in more detail, and we distinguish between the impact of the local virus situation and that of the impact and public perception of the national virus situation. We follow the approach of Chang and Meyerhoefer (2020), who study the effect of COVID-19 on demand for online grocery shopping services in Taiwan. They use data from Ubox, the largest agri-food e-commerce platform in Taiwan, and analyze the first three months of the COVID-19 pandemic (January 21-April 6 2020). We use data from the Dutch online-only supermarket Picnic and look at the first eight months of the pandemic.

We conclude that an additional hospital admission in a given Dutch municipality in a given week increases the number of unique visitors to the Picnic app by 7.3 percent, and drives up the sales per order with 0.31 percent. Moreover, more online keyword searches for COVID-19 lead to a decrease in the variety of ordered products and to people buying ‘more of the same’, which may indicate hoarding behavior. A one-unit change in the keyword search index increases Picnic’s app’s number of unique visitors at the municipality-week level by 12 and the sales per order by 19 cents.

In comparison, Chang and Meyerhoefer (2020) find that an additional confirmed case of COVID-19 increases the number of customers by 4.9 percent and sales by 5.7 percent. Similar to our results, they also find that online food shopping is highly responsive to national COVID-19 media coverage and online content.

This paper is organized as follows. Section 2 describes the data, our chosen measures of demand as well as the drivers of demand for online grocery shopping. Section 3 and 4 discuss the empirical model and the empirical results, respectively. Policy implications are given in Sect. 5.

## Data

We combine several datasets to test our hypotheses, namely transaction data from Picnic, a Dutch online-only supermarket, and data on the number of COVID-19 hospital admissions from the Dutch National Institute for Public Health and the Environment (RIVM). We also make use of Google search data. Picnic's customers are not fully representative of the Dutch population, as the supermarket does not operate in the three northern provinces of Drenthe, Friesland and Groningen. Nevertheless, the analysis based on Picnic's customer base provides a good picture of the effect of the COVID-19 pandemic on the purchasing behavior of Dutch consumers. As we analyze the data on a weekly basis, we automatically adjust for seasonal effects.

Picnic was founded in 2015. The online supermarket started in a medium-sized city in the Netherlands (Amersfoort) and had 4 electric delivery vans at the time. Now there are more than 1000 electric delivery vans driving around in some 120 Dutch towns.^2^ With hundreds of thousands of customers and a monthly expansion to new cities, Picnic is the second player on the Dutch market for online groceries in 2020. The largest player is Albert Heijn. The largest supermarket group in the Netherlands still held more than half (51.1 per cent) of the total in 2019, and that has dropped to 47.2 per cent by 2020. Picnic (19.8 per cent) narrowly overtook the second largest supermarket group Jumbo (19.7 per cent).^3^ Picnic is an online-only supermarket that is active only in cities, whereas the other two supermarkets get most of their business out traditional grocery shopping (in brick and mortar shops supermarkets) and are also active outside of cities when it comes to the delivery of groceries ordered online. According to market research agency GfK, the turnover of the online groceries market in 2020 has increased by 65 percent to 2.5 billion euros. Picnic saw its turnover increase by 95 percent in 2020 compared to the previous year.

### Measures of Demand

Our dependent variables of interest cover different dimensions of demand for online grocery shopping. They are Picnic's app's number of unique visitors, the sales per order and the commonality per order. Commonality is defined as the number of total items divided by the number of unique items in an order and is a measure of the variety of an order. An order consisting of five apples and five pears has a commonality of 5. A higher number means a lower variety.

We choose to include unique visitors as a measure of demand because waiting lists for Picnic's services could mean that latent demand is higher than demand as indicated, for example, by the sales per order. Due to supply constraints, e.g. limited delivery capacity, Picnic was not able to help all potential customers during the COVID-19 pandemic. We include commonality to measure the effect of COVID-19 on potential hoarding behavior. In the early days of the pandemic, many people stocked up on a number of non-perishable goods, suggesting that the variety of their order might be lower, and hence the commonality higher, than it would have been without COVID-19.

We construct the dependent variables at the municipality-week level using Picnic's microdata: the number of unique visitors is added up for each week and municipality, and sales per order and commonality are averaged for each week and municipality. We collect these data for the period from the first hospital admission in the Netherlands in the last week of February 2020 up until the end of August. This is our treated sample. We construct a control sample using the Picnic microdata for 2018 and 2019 during the same months, and the months of January and February 2020. Table 3 in the “Appendix” contains the descriptive statistics of the dependent variables for both periods.

**Fig. 1:**
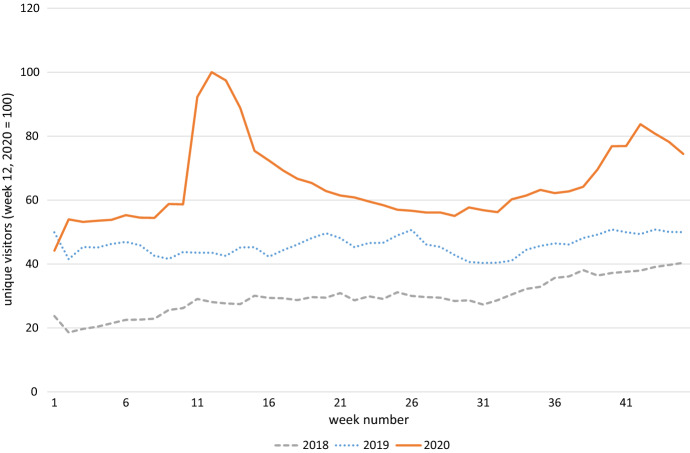
Demand for online groceries, measured by number of unique visitors

**Fig. 2:**
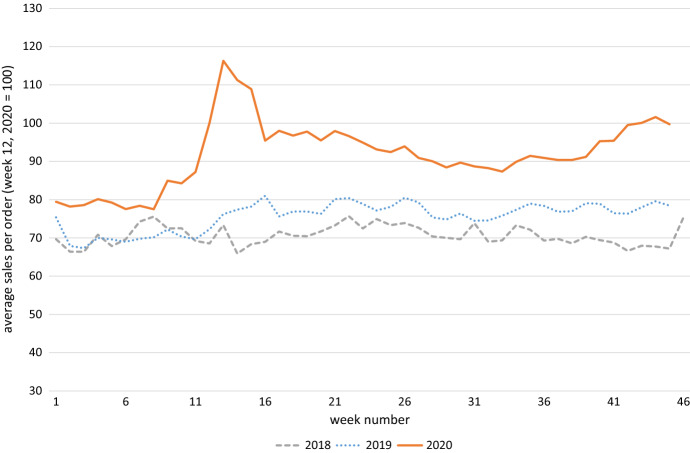
Demand for online groceries, measured by average sales per order

**Fig. 3:**
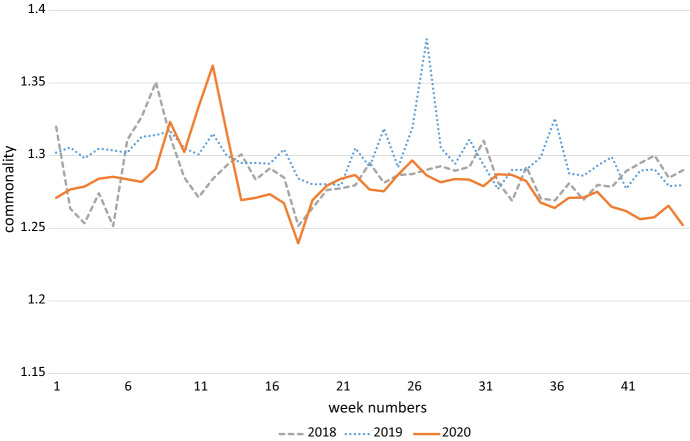
Demand for online groceries, measured by commonality

Figure 1, 2 and 3 show the aggregate development of the three dependent variables of interest for 2020, 2019 and 2018. Week numbers are on the x-axis. Figure 1 contains unique visitors, Fig. 2 average sales per order and Fig. 3 shows average order commonality. Unique visitors, and then sales rise sharply at the onset of the COVID-19 crisis, slowly abate, and then rise again at the start of the second wave. This pattern is not seen in 2018 and 2019, where the intra-year patterns are much more steady.

The development of average commonality in 2020, as seen in Fig. 3, is more difficult to distinguish from the other two years. Like unique visitors and sales, it does show a peak at the beginning of the sample period, but then slumps and does not pick up at the start of the second wave. Similar peaks and troughs are seen in the two earlier years at times, so it is difficult to attribute this visual finding to COVID-19.

### Drivers of Demand

Our independent variables of interest are the two variables that we hypothesize drive the increase in demand for online grocery shopping: the weekly COVID-19 hospital admissions at the municipal level and weekly Google keyword search data related to the COVID-19 pandemic at the national level.

Since testing in the Netherlands was limited during the first COVID-19 wave of infections, we use the number of local COVID-19 hospital admissions rather than the number of positive tests. This variable provides an indication to what extent consumers at the local level may be deterred from visiting physical stores due to worsening, local, COVID-19 conditions. Alternatively, it might also proxy for the number of people who are quarantining, and are reliant on online shopping for their groceries.

The Google keyword search data serve as a proxy for the spread of information about the virus at a national level and is invariant across municipalities. The index peaks at a value of 100 in week 13; all preceding and subsequent values are constructed in relation to that reference value. Together with the local hospital admissions data, this variable allows us to infer whether consumers base online shopping decisions on national news and virus conditions, or on local virus transmissions risks and realities.

As explained above, we construct a treated sample and a control sample. The treated sample covers the period end-of-February 2020 to August 2020. For the control sample, spanning the period January to August 2018 and 2019 and the first two months of 2020, there are zero COVID-19 hospital admissions and only a few keyword searches related to COVID-19. The lower two rows of Table 3 in the “Appendix” cover the descriptive statistics for these two independent variables: in the aggregate, and separately for the treated sample and the control sample.

**Fig. 4:**
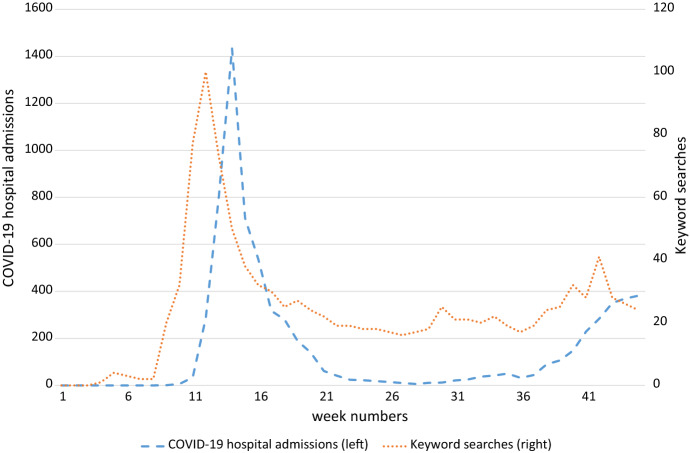
Drivers of demand for online grocery shopping over time

Figure 4 plots COVID-19 hospital admissions and keyword searches for the treated period, i.e. end-of-February to August 2020, with week numbers on the x-axis, where for illustrative purposes we aggregate COVID-19 hospital admissions to the national level. Since there are zero COVID-19 hospital admissions and keyword searches in 2018 and 2019, we only plot the data for 2020, so the week numbers on the x-axis refer to 2020.

There is a clear peak in both hospital admissions and COVID-19 keyword searches during the first wave, with hospital admissions slightly lagging keyword searches. Both number then drop and pick up again at the start of the second wave. This is similar to what we see happening in Fig. 1 and 2, where those two measures of demand sharply rise at the beginning of the sample, taper off and then increase again during the second wave.

## Empirical Model

We use a panel data model to gauge the effect of COVID-19 hospital admissions on online grocery shopping, in which the data from 2018, 2019 and the first two months of 2020 serve as the control group and data from the end-of-February 2020 onward as the treatment group. The first linear regression equation we use is specified as follows:

1 $$Y_{{jt}} = \alpha + \beta *COVID_{{jt}} + \gamma *search_{t} + t_{{year}} + t_{{week}} + m_{j} + \varepsilon _{{jt}}$$

where *Y*_*jt*_ is our outcome variable of interest, which is either the unique visitors to the Picnic app in municipality *j* (where *j* = 1, …., 83; we have 83 distinct municipalities in our data) and in week-year combination *t* (where *t* = 1, …, 135; our data contains 135 week-year combinations), the average sales per order, or the average commonality of the groceries ordered.

As for the independent variables, *α* is a constant, $COVID_{{jt}}$ is the number of hospital admissions in municipality *j* and in week-year combination *t* and $search_{t}$ is a national index of Google keyword searches in week-year combination *t*.^4^ We include fixed effects $t_{{year}}$, $t_{{week}}$  and $m_{j}$ to control for annual trends, seasonal effects and time-invariant differences between municipalities respectively, where *year* = 2018, 2019 and 2020, *week* = 1,…, 45 and *j* = 1, …, 83, for each of our 83 municipalities.

$\varepsilon _{{jt}}$ is an idiosyncratic error term for municipality *j* in week-year combination *t*.

We are also interested in the differential effect of hospital admissions for different regions: do the effects differ for urban versus non-urban areas? For that purpose we also estimate the following equation, with similar terms and subscripts as above:

2 $$Y_{{jt}} = \alpha + \beta *COVID_{{jt}} + \gamma *search_{t} + \delta *COVID_{{jt}} *urban_{j} + t_{{year}} + t_{{week}} + m_{j} + \varepsilon _{{jt}}$$

where $urban_{j}$ is a dummy indicating whether municipality *j* is urban or not, based on their population density.^5^

## Empirical Results

Table 1 contains the results of our analysis, where columns 1–3 show the results for Eq. 1 with different dependent variables, and columns 4–6 for Eq. 2.

**Table 1:** Effect of COVID-19 on demand for online shopping

	(1)	(2)	(3)	(4)	(5)	(6)
	Unique visitors	Sales per order	Commonality	Unique visitors	Sales per order	Commonality
COVID-19 hospital admissions	113.7***	0.162***	− 0.000498	− 0.636	0.549***	− 0.00183
	**(20.98)**	**(0.0586)**	(0.000445)	(24.06)	**(0.189)**	(0.00140)
Keyword searches	12.02***	0.190***	0.000650***	13.66***	0.184***	0.000675***
	**(3.289)**	**(0.0528)**	**(0.000204)**	**(2.941)**	**(0.0519)**	**(0.000199)**
COVID-19 hospital admissions*urban				117.0***	− 0.396**	0.00182
				**(33.50)**	**(0.168)**	(0.00121)
Total observations (N*T)	11,205	11,100	11,100	11,205	11,100	11,100
Municipalities (N)	83	83	83	83	83	83
Average week-year combinations per municipality (T)	135	133.7	133.7	135	133.7	133.7
Total number of explanatory variables	130	130	130	131	131	131
Within R-squared	0.402	0.411	0.015	0.408	0.412	0.015
Between R-squared	0.525	0.003	0.023	0.529	0.002	0.007

Standard errors are clustered at the municipality and week level. All models include municipality, week and year fixed effects. The total number of explanatory variables includes the continuous explanatory variables, the municipality, week and year fixed effects and a constant

Results in bold are significant at at least the 10% level

***p < 0.01, *p < 0.5

Models 1 and 2 show that both local COVID-19 hospital admissions and national-level keyword searches are related to demand for online grocery shopping.

An additional hospital admission in a given municipality in a given week increases the number of unique visitors by 114 persons, or 7.3 percent, and drives up the sales per order with 16 cents, or 0.31 percent.^6^ Model 3 shows there is no effect of the number of hospital admissions at the local level on the commonality of orders; the variety of ordered products within a given order does not change. Since we hypothesize that a decrease in variety is a sign of hoarding, it appears that local hospital admissions do not cause consumers to hoard.

Search behavior also appears to be an additional predictor for the expenditure per customer and the number of unique visitors at municipal level, as shown in model 1 and 2. The sales per order increases by 19 cents when the keyword search index shows a one-unit increase. The keyword web search index runs from 0 to 100, and we see that a one-unit change increases the number of unique visitors at the municipality-week level by 12. Also, search behavior does appear to influence the variety of ordered groceries, as model 3 shows.^7^ Additional information seeking at the national level increases the commonality and reduces the variety of grocery shopping. This might reflect the panic buying seen at the beginning of the first wave, which coincided with the peak in Google search activity seen in Fig. 3. As more Google searches for corona-related terms, the variety of messages decreases and people buy ‘more of the same’.

Online purchasing behavior is thus determined both by the perception of the national virus situation and by the number of local hospital admissions—but the hoarding behavior of consumers is only influenced by the national perception.

### Differences in Demand Between Urban and Non-Urban Municipalities

Models 4 through 6 in Table 1 include an interaction effect between an urbanization dummy and local hospital admissions. Model 4 shows that in urban municipalities the effect of hospital admissions on unique visitors is larger, whereas model 5 shows that the effect on sales per order is smaller. It appears that consumers in urban and non-urban localities respond slightly different to local COVID-19 hospital admissions.^8^

There are many possible explanations for this. Perhaps Picnic’s advertising is more effective with or targeted toward urban consumers, making it more likely for them to visit the app. Perhaps they put in more orders, but spend less per order, as compared to non-urban consumers, who might buy more in bulk.^9^

Model 6 does not really differ from model 3. There is an effect of keyword searches on commonality. The effect of hospital admissions, meanwhile, is not significant, neither for urban, nor for non-urban consumers.

### Robustness Checks

By including several different fixed effects, we ensure that our results are more robust than they otherwise would be, but we still cannot exclude the possibility of spurious correlation. Following Chang and Meyerhoefer (2020), we therefore conduct a falsification test, where we regress the three main demand measures for the non-COVID period on local hospital admissions for the COVID period, to validate our results.^10^ Table 2 shows that there is no effect of hospital admissions on unique visitors or sales per order when we run this falsification test. Hence it is more likely that the positive trend in 2020 we see is driven by COVID-19 hospital admissions. This falsification test is not a perfect way to exclude spurious correlation. It sometimes produces false positives: in this case, the coefficient of hospital admissions is significant at the ten percent level. This correlation is in itself spurious, since hospital admissions from 2018 and 2019 are combined with commonality statistics from 2020.

**Table 2:** Falsification test of the effect of hospital admissions on demand for online grocery shopping, using only the non-COVID sample

	(1)	(2)	(3)
	Unique visitors	Sales per order	Commonality
COVID-19 hospital admissions	3.866	0.00295	− 0.000242*
	(5.282)	(0.0109)	**(0.000128)**
Total observations (N*T)	6142	6081	6081
Municipalities (N)	83	83	83
Average week-year combinations per municipality (T)	74	73.3	73.3
Total number of variables	120	120	120
Within R-squared	0.151	0.100	0.012
Between R-squared	0.446	0.006	0.001

Standard errors are clustered at the municipality and week level. All models include municipality, week and year fixed effects. The total number of explanatory variables includes the continuous explanatory variables, the municipality, week and year fixed effects and a constant

Results in bold are significant at at least the 10% level

*p < 0.1

## Discussion and Policy Implications

Since COVID-19, people have started shopping online more frequently. By analyzing data from a Dutch online supermarket, we show that both local and national COVID-19 conditions have affected demand for online food shopping, a finding confirmed in other studies as well. In the Netherlands, an additional, local hospital admission increased app traffic by 7.3 percent. The effect on sales per order was smaller, at 0.31 percent, but still significant. Considering the large total number of orders, this generally adds up to a significant revenue boost for online food retailers. Online search behavior, meanwhile, correlates with these measures but also with the composition of orders, affecting the variety of groceries bought.

A major question is how persistent this increase in demand will be and what would have happened had COVID-19 not taken place. The online supermarket we study was on a strong growth path in 2018 and 2019, prior to COVID-19, so it is difficult to disentangle how much of the growth in 2020 is due to structural factors and how much is due to COVID-19.

It does appear that in 2020 growth prior to COVID-19 was slowing somewhat compared to the same period in 2018 and 2019. Growth of the number of unique visitors in weeks 2–8 was 23 percent in 2018, 3 percent in 2019, and only 1 percent in 2020.^11^ The respective numbers for sales per order are 14 percent, 3 percent and roughly zero percent.

After week 8 in 2020, the number of unique visitors and sales per order picked up. Between that moment and week 28, for example. When hospitalizations were at or near zero, sales increased by 16 percent. The growth in sales per order is higher than during the same period in 2018 and 2019, when it was zero and 8 percent respectively. And for the week 2–45 period, which is our full sample, sales per order are up by 28 percent. This implies that for the COVID-19 period at least, a significant behavioral change has taken place. More data measured at a later moment should help determine whether sales per order will return to the plateau they appeared to have reached in the 2020 pre-COVID weeks, remain the same as now, or increase further. A survey conducted by market research firm Nielsen on the Dutch online groceries shopping market seems to indicate that customers will continue to buy their food online, also after the Covid-19 crisis.^12^

Our findings, which document a strong behavioral response to COVID-19, have policy implications both in Netherlands and elsewhere. An acute recommendation concerns the following: online shopping helps minimize transmission risk. This suggests a need for active government policy to stimulate online grocery shopping during this final stretch of the pandemic. Interestingly, there have been numerous proposals to impose additional taxes on online grocers, as they have seen large increases in business over the past year, while other businesses have floundered or have gone under. The positive externality of fewer infections suggests that such tax policies should at least wait until the pandemic is over, and governments in the meantime might even want to consider ways to boost online shopping.

More generally, and in the longer run, a shift to online shopping generates significant additional consumer surplus. This is due to an increase in the variety of supply and greater convenience (Dolfen et al., 2019). To allow as many people as possible to benefit from this, government policies should be aimed at ensuring that as many households as possible have access to sufficiently fast and stable internet and are taught the digital skills needed to shop and pay online. Looking at the Eurostat data (2020) show that this is not necessary in countries like the Netherlands, Germany, the Scandinavian countries, Estonia, Slovenia, Austria, Belgium, France and Spain but governments in other European countries are well-advised, for example, to broaden access to internet and enable people to engage in e-commerce, including online grocery shopping. Internet penetration in the Netherlands is already high at 98 percent of households, but it is low in parts of Eastern Europe, the South of Europe and elsewhere in the world. The European Union already has a program to further roll out broadband in the member states. Because the proportion of individuals aged 16 to 74 in the EU-27 who ordered or bought goods or services online for private use, is remarkably low in countries like Bulgaria and Romania (< 25%) and in Greece, Portugal and Italy (< 40%), governments in those countries could link this program to an educational program to increase citizens' digital skills. European banks can also help by accelerating the transition to digital payments by their customers, which is necessary for online shopping.

### Supplementary Information

Below is the link to the electronic supplementary material.

Supplementary file1 (XLSX 36 kb)

## Appendix

See Table 3.

**Table 3:** Descriptive statistics

Sample		Full sample			Treated sample (COVID-19 period)			Control sample (non- COVID-19 period)		
Variable	Definition	Mean	SD	N × T	Mean	SD	N × T	Mean	SD	N × T
Commonality	Average of grocery items divided by unique grocery items per order	1.289	0.177	11,100	1.281	0.051	3069	1.292	0.206	8031
Local hospital admissions	Number of municipality-level hospital admissions	0.640	4.267	11,205	2.335	7.905	3071	0	0	8134
Keyword searches	Search index value where value = 100 at peak	8.119	16.05	11,205	29.30	17.91	3071	0.122	0.576	8134

## Footnotes

1 Cavallo not only shows that food consumption increased during the pandemic, but he also shows that this has an effect on inflation. Because food became relatively more expensive and households (especially lower-income households) spend a larger share of their income on food during the COVID-19 crisis, COVID-19 inflation is higher than the ordinary CPI.

2 https://picnic.app/nl/over-picnic/ In Dutch, 2021.

3 https://www.businessinsider.nl/online-boodschappenmarkt-ah-jumbo-picnic/ In Dutch, February 17 2021.

4 This index captures searches for the terms ‘corona’ and ‘COVID’.

5 We use the 5-point scale of urban density as defined by Statistics Netherlands. We classify all municipalities with a score of 1 or 2 as urban, and others as non-urban.

6 The marginal effects in percentage terms are evaluated at the mean of the control sample.

7 The absolute size of the commonality coefficient appears small, but average commonality consistently fluctuates between 1.25 and 1.35. During the early period of COVID-19, as panic buying ensued, average commonality rose from 1.29 in week 8 to a high of 1.36 in week 12. A 100-unit increase in the keyword search index raises commonality by 0.065, ceteris paribus.

8 There might also simply be more visitors in urban municipalities because there are more people living there. We tested this by merging our data with municipality-level population data then expressed unique visitors in relative terms. The direction and significance of the effect of local hospital admissions remains the same.

9 Note that the decision to buy in bulk is not affected by delivery fees of any kind. Picnic does not charge a delivery fee.

10 This means that in this case we regress our demand measures for week 9 and later in 2018 and 2019 on hospital admissions for week 9 and later in 2020, as hospital admissions are zero in week 1–8 in 2020.

11 We use week 2 as our starting week because there seems to be a beginning-of-year effect (an uptick) in the first week in every year in our sample.

12 https://retailtrends.nl/news/60413/populariteit-online-boodschappen-houdt-ook-na-de-crisis-aan May 14 2020. In Dutch.
